# Measuring biomarkers of acute kidney injury during renal replacement therapy: wisdom or folly?

**DOI:** 10.1186/cc13933

**Published:** 2014-06-19

**Authors:** Marlies Ostermann, Lui G Forni

**Affiliations:** 1 Department of Critical Care & Nephrology, Guy’s & St Thomas’ Hospital, Westminster Bridge Road, London SE1 9RT, United Kingdom; 2 Department of Critical Care, Western Sussex Foundation Hospitals Trust, Lyndhurst Road, Worthing, West Sussex BN11 2DH, United Kingdom; 3 Brighton & Sussex Medical School, Falmer, Brighton BN1 9PX, United Kingdom

## Abstract

Early data are now appearing relating to the measurement of biomarkers of acute kidney injury during renal replacement therapy. These data go some way in describing the clearance of these molecules during renal support. Understanding the potential clearance, or otherwise, of these proteins may lead to directing our therapies in the future particularly with regard to cessation of renal support. We describe a recent study which has provided data that may aid in addressing this issue.

## 

Acute kidney injury (AKI) remains a frequent complication of critical illness. The limitations of both the serum creatinine and the urine output in alerting the clinician to renal injury have catalyzed the growing body of research examining candidate molecules which may provide an earlier signal highlighting the presence of renal injury. The study by Schilder and colleagues [1] in the previous issue of *Critical Care* examines one of the more popular markers of AKI, namely neutrophil gelatinase-associated lipocalin (NGAL), but rather than writing another article on the accuracy of NGAL to diagnose AKI, the authors focus on the effects of renal replacement therapy (RRT) on NGAL levels.

NGAL is a member of the lipocalin family of proteins that transport small hydrophobic ligands and has numerous roles in inflammatory processes as well as cancer [2]. NGAL expression has been reported in many tissues where it may provide protection against bacterial infection and modulate oxidative stress. Moreover, it shows some promise as a potential biomarker for the early diagnosis of AKI along with other potential candidates [3-5]. So given that NGAL may herald AKI, why is this study of interest? One could argue that employment of RRT already implies renal injury and hence knowledge of its occurrence is somewhat superfluous. There is evidence that, in addition to helping to diagnose AKI earlier, single NGAL levels can help to predict outcome (that is, severity of AKI, need for RRT, and mortality) [4-6]. However, Zeng and colleagues [7] measured urinary NGAL levels in 199 patients undergoing surgery pre-operatively and regularly until day 14 post-surgery and found that serial NGAL levels were poor predictors for renal recovery after AKI had occurred. Defining renal recovery in patients receiving RRT is particularly challenging. Urine output may aid the clinician but often is confounded by the use of diuretics, and serum creatinine is of little value. The specific role of novel biomarkers in predicting successful discontinuation of RRT has not been studied but would require confirmation that the biomarker (or panel of biomarkers) is not cleared during RRT.

Schilder and colleagues measured serial NGAL levels in serum and ultrafiltrate for 9 hours in 42 patients on RRT with particular attention to volume balance and delivered dose. A biocompatible cellulose triacetate hemofilter with a cutoff of approximately 40 kDa was used, and the sieving coefficient (SC) for NGAL was determined. The SC is the ratio of the concentration of solutes in the ultrafiltrate to that of the plasma. Thus, an SC of 1 describes complete permeability whereas an SC of 0 implies complete impermeability. Importantly, SC not only is driven by the molecular weight/size of the solute but also is dependent on protein binding and porosity of the filter. NGAL has a molecular weight of 25 kDa; thus, one may expect it to be relatively easily filtered. If that were to be the case, then monitoring of NGAL levels during RRT would tell us little save an approximation to adequacy! What was observed was no difference between NGAL levels pre- and post-filter with a relatively low SC of between 0.2 and 0.4 and a decrease of the observed SC with time. Certainly, there is no evidence from this study that RRT affects plasma NGAL levels in any significant manner. The relatively low SC probably reflects a degree of protein binding as it is well known to bind with other proteins such as bacterial siderophores. Indeed, this study confirms an earlier, albeit smaller, study on NGAL clearance which used a polysulphone filter and similarly observed little impact on SC values [8]. It should be borne in mind, however, that the SC profile differs between filters as does the homogeneity of pore size. Interestingly, the only other study to use an experimental hemofiltration set-up with a polysuphone filter gave calculated SC values of 0.2 to 0.4 [9]. In that study, unlike the one by Schilder and colleagues, SC values increased with time, and this increase was attributed to membrane adsorption. No significant differences were observed with differing anticoagulants, although the observed concentrations of NGAL in the ultrafiltrate of those treated with citrate showed a trend toward lower concentrations which may reflect less generated NGAL from sequestered inflammatory cells.

So how will this study change our practice? At this juncture, in truth, probably not at all, but it does open the door to future studies. It is clearly important, when evaluating the role of any AKI biomarker to guide therapy, including tailoring or discontinuing RRT, to confirm that the biomarker is not removed by the treatment itself. In the future, identification of a particular marker (either a single value or trends) which indicates renal recovery while on RRT may allow timely discontinuation and prevent unnecessary exposure to extracorporeal circuits. Clearly, at present, we have no tools to allow us to do this, but this study does provide some potential. As we are aware, predicting anything in the intensive care unit environment is difficult, especially - as Niels Bohr famously said - if it’s about the future! Hopefully, in the not too distant future, patients may benefit from the predictive tools we are yet to discover.

## Abbreviations

AKI: acute kidney injury; NGAL: neutrophil gelatinase-associated lipocalin; RRT: renal replacement therapy; SC: sieving coefficient.

## Competing interests

The authors declare that they have no competing interests.
